# Metabolic Dysfunction‐Associated Steatotic Liver Disease (MASLD) Impacts Long‐Term Outcomes After Curative‐Intent Surgery for Hepatocellular Carcinoma

**DOI:** 10.1111/apt.70002

**Published:** 2025-02-18

**Authors:** Deniz Uluk, Justus Pein, Sophia Herda, Frederik Schliephake, Carolin V. Schneider, Jude Bitar, Katharina Dreher, Dennis Eurich, Ingrid W. Zhang, Lukas Schaffrath, Timo A. Auer, Federico Collettini, Cornelius Engelmann, Frank Tacke, Johann Pratschke, Isabella Lurje, Georg Lurje

**Affiliations:** ^1^ Department of Surgery, Campus Charité Mitte, Campus Virchow Klinikum Charité‐Universitätsmedizin Berlin Berlin Germany; ^2^ Department of General, Visceral and Transplantation Surgery Heidelberg University Hospital Heidelberg Germany; ^3^ Department of Medicine III University Hospital RWTH Aachen Aachen Germany; ^4^ Department of Gastroenterology and Hepatology, Campus Charité Mitte, Campus Virchow Klinikum Charité‐Universitätsmedizin Berlin Berlin Germany; ^5^ Department of Radiology Charité – Universitätsmedizin Berlin Berlin Germany

## Abstract

**Background:**

Curative surgery for hepatocellular carcinoma (HCC) includes liver resection (LR) and orthotopic liver transplantation (OLT). Due to the obesity epidemic, metabolic dysfunction‐associated steatotic liver disease (MASLD) is a frequent HCC aetiology that often coincides with increased alcohol consumption, termed MetALD, or even alcohol‐associated liver disease (ALD).

**Methods:**

Patients undergoing LR or OLT for HCC at Charité—Universitätsmedizin Berlin (2010–2020) were included in this retrospective cohort study investigating disease aetiology, time to recurrence (TTR), overall survival (OS) and CT‐based body composition.

**Results:**

Out of 579 patients with HCC, 417 underwent LR and 162 OLT. Tumour aetiologies were viral *n* = 191 (33.0%), MASLD *n* = 158 (27.3%), MetALD *n* = 51 (8.8%), ALD *n* = 68 (11.7%) and other/cryptogenic *n* = 111 (19.2%). Patients with MASLD and MetALD had more intramuscular (*p* < 0.001, *p =* 0.015) and visceral fat (both *p* < 0.001) than patients with non‐metabolic dysfunction aetiologies. Patients with MASLD‐HCC had comparable TTR (median 26 months, [95% CI: 23–31] vs. 30 months [95% CI: 4–57], *p* = 0.425) but shorter OS than patients with other HCC aetiologies (63 months [95% CI: 42–84] vs. 80 months [95% CI: 60–100], hazard ratio: 1.53 [95% CI: 1.050–2.229], *p* = 0.026) after LR. Multivariate analysis confirmed MASLD aetiology, portal vein thrombosis and MELD score ≥ 10 as independent prognostic factors for OS in LR (adjusted *p* = 0.021,*p* < 0.001,*p =* 0.003), even after excluding in‐hospital mortality (adjusted *p* = 0.016,*p* = 0.002,*p* = 0.002). Causes of death were similar in MASLD and non‐MASLD aetiology.

**Conclusions:**

Patients with HCC undergoing LR and meeting the new MASLD criteria have significantly shorter OS. This study provides empirical prognostic evidence for the novel MASLD/MetALD classification in a large European cohort of patients undergoing curative‐intent HCC therapy.

## Graphical abstract

Patients with HCC undergoing liver resection and meeting the new MASLD and MetALD criteria have characteristic alterations of body composition. Patients with MASLD‐HCC have similar disease‐free survival but significantly shorter OS than other HCC aetiologies after curative‐intent therapy.

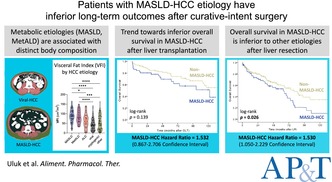

## 1 Introduction

Hepatocellular carcinoma (HCC) is the most frequent primary liver cancer and one of the leading causes of cancer‐related death, with incidences rising worldwide [1]. Due to the late clinical presentation of HCC, the severity of the underlying liver disease and frequently impaired overall condition of the patient, only 15% are amenable to curative‐intent surgery at the time of diagnosis [2, 3]. Surgical liver resection (LR) and orthotopic liver transplantation (OLT) constitute the mainstay of curative HCC treatment, together with ablative procedures for smaller tumours, with the possibility to bridge patients to transplantation. The standard of care for patients with intermediate‐stage HCC is transarterial chemoembolisation (TACE), and systemic therapy at intermediate or advanced stages, with significantly shorter survival than that after curative‐intent therapy [4, 5].

Hepatocellular carcinoma typically develops in chronic liver disease, with fibrosis and/or steatosis [6, 7]. Over 80% of HCCs arise in a manifest liver cirrhosis [8]. While chronic viral hepatitis‐induced liver cirrhosis is the most frequent HCC aetiology worldwide, the burden of metabolic syndrome and steatotic liver disease, its hepatic manifestation, is increasing [1, 9]. As such, current estimates attribute one‐half of cirrhosis‐associated and over one‐third of HCC‐associated deaths in the US to steatotic liver disease [10].

A new classification of non‐alcoholic fatty liver disease (NAFLD) was recently endorsed by hepatological societies, combining the presence of liver steatosis with the presence of cardiometabolic risk factors [11]. In this context, a new category, termed metabolic liver disease with moderately increased alcohol intake (MetALD), was put forward to identify individuals with metabolic dysfunction‐associated steatotic liver disease (MASLD) who consume greater amounts of alcohol regularly [11] but do not exceed the gender‐specific cutoffs for the diagnosis of alcohol‐associated liver disease (ALD). As a result, the classification provides a more detailed understanding of patients' metabolic status and an additional assessment of their cardiometabolic health. Current treatment algorithms in the curative‐intent setting do not distinguish between different HCC aetiologies, and most prognostic studies do not afford a granular analysis of the underlying steatotic liver disease—with viral versus non‐viral disease most prevalently reported [12].

Changes of body composition are highly frequent in patients with progressive liver disease, while the dysfunction of the muscle and lipid compartments can reciprocally contribute to metabolic and steatotic liver injury [13]. A long‐recognised, systemic pathology in patients with cirrhosis is a catabolic state with a loss of muscle mass and strength, coined sarcopenia [14]. In the context of rapidly rising obesity rates and MASLD, the different fat compartments have a newfound relevance in the end‐stage liver disease [15]. While subcutaneous fat is the most obvious fat compartment upon clinical examination, visceral and hepatic fat are metabolically interconnected and associated with cardiovascular morbidity [16]. Intramuscular fat accumulation, termed myosteatosis, is associated with decreased metabolic health [17], inferior muscle function and a reduced ability for muscular glucose uptake and metabolism [13]. Thus, body composition, defined as the quality and quantity of muscle and the quantity and distribution of fat, can provide deeper insights into the metabolic status of patients with HCC and their underlying liver disease.

Here, we explored the novel MASLD and MetALD classifications in patients undergoing curative‐intent HCC surgery, reinforced with body composition analyses. We evaluated the applicability of the MASLD and MetALD categories to retrospective patient data. Furthermore, we examined the relationship between the underlying liver disease and pathologies in the CT‐based quality and quantity of muscle and fat distribution in the context of the updated classification of HCC aetiology in a large European cohort of patients with HCC undergoing curative‐intent therapies.

## 2 Methods

### 2.1 Patients and Treatment

All consecutive patients with localised HCC undergoing curative‐intent LR or OLT at the Department of Surgery, Charité—Universitätsmedizin Berlin, from 2010 to 2020 were included in this study. This study was conducted in accordance with the Declaration of Helsinki, the good clinical practice (ICH‐GCP) guidelines, and was approved by the institutional review board of the Charité—Universitätsmedizin Berlin, Berlin, Germany in 2021 (EA1/105/21). Informed consent was waived by the committee at study approval, based on the retrospective, pseudonymised analysis of available clinical patient data and the anonymised reporting of patient characteristics that did not allow retrospective patient identification.

Patients diagnosed with localised, non‐metastatic HCC underwent cross‐sectional computed tomography (CT) and/or magnetic resonance imaging (MRI), histopathological assessment and evaluation of liver function and disease. The decision between LR and OLT considered tumour burden and location, the degree of liver disease, predictors of recurrence (multifocality, vascular invasion and satellite nodules) and individual considerations [18] and was made by an interdisciplinary tumour and transplantation board. Patients with small, solitary tumours were also considered for ablation. Patients within Milan criteria received standard exception for HCC (SE‐HCC) points on the waiting list, while patients whose tumour burden exceeded Milan criteria were wait‐listed without SE‐HCC Model for End‐Stage Liver Disease (MELD) points, according to Eurotransplant and national regulations [19]. Patients requiring extensive resections, causing an insufficient future liver remnant, underwent preoperative portal venous embolisation (PVE), and in selected cases of PVE failure, additional parenchymal transection (associated liver partition with portal vein ligation for staged hepatectomy, ALPPS). For survival analyses, laboratory MELD score was dichotomised at ≥ 10 points, as described in previous HCC cohorts undergoing resection and transplantation [20].

The retrospective identification of MASLD aetiology was made according to the 2023 consensus [11], as histology of liver steatosis, combined with at least one of the following cardiometabolic risk factors: Impaired glucose tolerance/type II diabetes, arterial hypertension, dyslipidemia and overweight/obesity (BMI ≥ 25 kg/m^2^). The severity of the underlying liver disease in the non‐tumorous area was staged with the Desmet fibrosis score from F0 (no fibrosis) to F4 (cirrhosis) [21] and the percentage of steatosis (stage 0: steatosis in < 5% of hepatocytes; stage 1: steatosis in > 5% and < ^1^/_3_ of hepatocytes; stage 2: steatosis in ^1^/_3_ to ^2^/_3_ of hepatocytes; stage 3: steatosis in ≥ ^2^/_3_ of hepatocytes).

**FIGURE 1:**
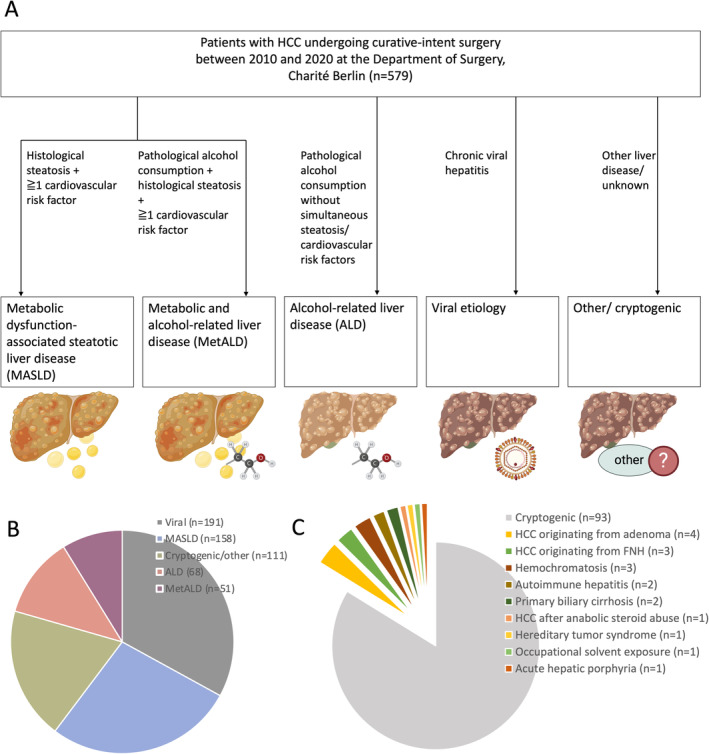
Definition and incidence of HCC aetiologies. (A) Flowchart of the definition of HCC aetiology, adapted from the 2023 Delphi consensus statement [11]. Retrospective quantification of alcohol consumption was considered inaccurate, and therefore patients with relevant alcohol consumption were differentiated by their incidence of steatosis and cardiovascular risk factors. (B) Tumour aetiologies of the overall patient cohort. (C) Tumour aetiologies of the cryptogenic/other group. Panel 1A was created with Biorender.com.

Patients wait‐listed for OLT and underlying ALD required a period of > 6 months of biochemically proven sobriety, according to Eurotransplant and national consensus [22]. In the LR group, ALD was evaluated from self‐reported consumption at the time of operation. A retrospective quantification of alcohol consumption was not possible to differentiate the nuanced aspects of the new MetALD group from the Delphi consensus statement [11]. This classification states that MetALD (besides having steatosis and a cardiometabolic risk factor) is classified as a daily alcohol intake of 20–50 g/d for women and 30–60 g/d for men, while alcohol consumption exceeding these values is ALD, regardless of cardiometobolic risk factors [11]. In our retrospective approach, if patients met MASLD criteria and had self‐reported alcohol consumption exceeding 20 g in females and 30 g in males, patients were categorised as either MetALD if they had steatosis and a cardiometabolic risk factor or as ALD in the absence of steatosis or cardiometabolic risk factors (Figure 1A). Long‐term outcomes in this study were analysed separately by the therapeutic approach, LR and OLT, because it is recognised that patients after LR have much higher recurrence rates and shorter survival compared to OLT [23].

Oncological and overall follow‐up was provided by the Department of Surgery and the Department of Gastroenterology and Hepatology outpatient clinics of the Charité—Universitätsmedizin Berlin, as well as by community‐based hepatologists.

### 2.2 Body Composition

Body composition was analysed in patients undergoing LR, with abdominal CT scans conducted within 100 days before surgery, as previously described [15]. Images were exported at the level of the 3rd lumbar vertebra and analysed semi‐automatically with the workstation SlicerCIP extension and body composition module of the 3D Slicer software (version 4.10.2) [24]. Standardised cutoffs (skeletal muscle −29 to 150 Hounsfield units (HU), visceral fat −150 to −50 HU, subcutaneous fat −190 to −30 HU) were used to calculate the cross‐sectional area of the respective compartment in cm^2^ [25]. Visceral and subcutaneous adipose tissues were defined as areas of fat inside and outside of the abdominal wall, respectively. Normalisation to patients' stature yielded the parameters visceral fat index (VFI), subcutaneous fat index (SFI) and skeletal muscle index (SMI), in cm^2^/m^2^.

Sarcopenia was defined according to previously reported scores in end‐stage liver disease, as SMI < 39 cm^2^/m^2^ in female and < 50 cm^2^/m^2^ in male patients [26]. Sarcopenic obesity was defined as sarcopenia in the presence of BMI ≥ 25 kg/m^2^ (overweight/obesity), as in previous oncological cohorts [27]. Myosteatosis, reflective of fat infiltration in muscles, was defined as skeletal muscle radiation attenuation (SM‐RA) < 41 HU for patients with BMI < 25 kg/m^2^ and < 33 HU for patients with BMI ≥ 25 kg/m^2^ [28]. Visceral obesity was defined as VFA ≥ 100 cm^2^. For subcutaneous obesity, SFI was dichotomised at the upper tertile of the cohort (72.04 cm^2^/m^2^), as described previously [29].

### 2.3 Statistical Analysis

Independence between categorical variables was assessed with the chi‐squared test and given for two‐sided testing. Continuous variables were reported as median and range and analysed using the Mann–Whitney *U* test. Time to recurrence (TTR) was defined as the period between operation and recurrence or the last contact in patients without recurrence. Overall survival (OS) was defined as time from surgery until death or until last contact if patients were alive. The impact of clinical and body composition parameters on TTR and OS was analysed with uni‐ and multivariable logistic regression analyses. Parameters significant in the univariable analysis were included in the multivariable analysis, under exclusion of variables with suspected collinearity (e.g., to avoid multicollinearity with the UICC stage, tumour size, T stage and vascular invasion were omitted from the multivariable models). Post‐operative survival was visualised with Kaplan–Meier curves, with time given in estimated medians with 95% confidence intervals (CI) and hazard ratios (HR). Statistics were computed with SPSS Statistics 24 (IBM Corp., Armonk, NY) and GraphPad Prism 9 (GraphPad Software, Boston, MA). The level of significance was set to *p* < 0.05.

## 3 Results

Between January 2010 and November 2020, a total of 579 patients with HCC underwent curative‐intent LR (*n* = 417, 72%) or OLT (*n* = 162, 28%). Most patients (*n* = 447, 77%) were male. The HCC cohort had a high prevalence of cardiometabolic risk factors, such as type 2 diabetes (*n* = 247, 43%), arterial hypertension, (*n* = 456, 79%) and dyslipidemia (*n* = 111, 30%). A total of 191 patients (33%) had viral tumour aetiology, of which 69/191 (36%) patients had chronic hepatitis B, and 113/191 (59%) hepatitis C, while 9/191 (5%) patients were co‐infected with both hepatitis B and C virus. Of 191 patients with chronic hepatitis, 66 (35%) were considered cured at the time of surgery, 68 (35.6%) had chronically active disease with an above‐threshold viral load, while in 57 (30%) of patients, the viral load was not assessed at the time of surgery. In 158 patients (27%), the HCC aetiology was MASLD, 51 patients (9%) had MetALD, 68 patients (12%) had ALD and 111 patients (19%) were classified as cryptogenic/other (Figure 1B,C). Most of the cohort had early tumour stages, with 85% of patients (*n* = 496) diagnosed with T1 or T2 stages. Patients undergoing OLT had a median LabMELD score of 12 (range: 5–40), with 126 (78%) cases within the Milan criteria (Table 1).

**TABLE 1:** Clinical and perioperative characteristics.

Patient characteristics	All patients (*n* = 579)	Liver resection (*n* = 417)	Orthotopic liver transplantation (*n* = 162)
Age (years)	65 (21–88)	68 (21–88)	61 (41–78)
Sex ratio (female: male), *n* (%)	132 (22.8): 447 (77.2)	107 (25.7): 310 (74.3)	25 (15.4): 137 (84.6)
Aetiology, *n* (%)			
Viral	191 (33.0)	128 (30.7)	63 (38.9)
MASLD	158 (27.3)	107 (25.7)	51 (31.5)
MetALD	51 (8.8)	51 (12.2)	
ALD^a^	68 (11.7)	28 (6.7)	40 (24.7)
Cryptogenic/other	111 (19.2)	103 (24.7)	8 (4.9)
Metabolic dysfunction, *n* (%)			
Diabetes mellitus type II	247 (42.7)	161 (38.6)	86 (53.1)
Impaired glucose tolerance	9 (1.6)	5 (1.2)	4 (2.5)
Arterial hypertension	456 (78.8)	327 (78.4)	129 (79.6)
Dyslipidemia	172 (29.7)	134 (32.1)	38 (23.5)
Surgical proceDeniz Ulukre, *n* (%)			
Portal vein embolisation	23 (4.0)	23 (5.5)	n.a.
In situ split	1 (0.2)	1 (0.2)	n.a.
Lymphadenectomy	135 (23.8)	124 (30.6)	11 (6.8)
Vascular replacement	4 (0.7)	4 (1.0)	n.a.
Additional RFA	5 (0.9)	5 (1.2)	n.a.
Additional MWA	5 (0.9)	5 (1.2)	n.a.
Operative approach, *n* (%)			
Conventional	421 (72.7)	259 (62.1)	162 (100.0)
Laparoscopic	127 (21.9)	127 (30.5)	0
Robotic	31 (5.4)	31 (7.4)	0
Number of resected segments^b^	4 (1–8)	2 (1–7)	8 (4–8)
HCC within Milan, *n* (%)	292 (50.4)	166 (39.8)	126 (77.8)
T category, *n* (%)			
T0^b^	4 (0.7)	0	4 (2.5)
T1	307 (53.0)	227 (54.4)	80 (49.4)
T2	185 (32.0)	118 (28.3)	67 (43.4)
T3	63 (10.9)	54 (12.9)	9 (5.6)
T4	11 (1.9)	11 (2.6)	0
Largest tumour diameter (mm)^b^	39 (0–240)	50 (7–240)	24 (0–135)
Nodal positivity, *n* (%)	11 (1.9)	11 (2.6)	0
R category, *n* (%)			
R0	524 (92.4)	363 (89.4)	162 (100.0)
R1	43 (7.6)	43 (10.6)	0
(Micro‐)vascular invasion, *n* (%)	117 (20.3)	102 (24.6)	15 (9.3)
Lymphovascular invasion, *n* (%)	28 (4.9)	25 (6.1)	3 (1.9)
Perineural invasion, *n* (%)	5 (0.9)	5 (1.2)	0
Tumour grading, *n* (%)			
G0^b^	3 (2.0)	0	3 (2.0)
G1	70 (12.9)	40 (10.2)	30 (20.4)
G2	365 (67.5)	277 (70.3)	88 (59.9)
G3	102 (18.9)	77 (19.5)	25 (17.0)
G4	1 (0.2)	0	1 (0.7)
Tumour stage, AJCC/UICC (8th ed), *n* (%)			
0^b^	4 (0.7)	0	4 (2.5)
I	317 (54.7)	238 (57.1)	79 (49.1)
II	174 (30.2)	105 (25.3)	69 (42.9)
IIIa	25 (4.3)	18 (4.3)	7 (4.3)
IIIb	34 (5.9)	32 (7.7)	2 (1.2)
IIIc	8 (1.4)	8 (1.9)	0
IVa	9 (1.6)	9 (2.2)	0
IVb	5 (0.9)	5 (1.2)	0

*Note:* Data presented as median and range if not noted otherwise.

Abbreviations: AJCC, American Joint Committee on Cancer; BMI, body mass index; G, Grade; HCC, hepatocellular carcinoma; MASLD, metabolic dysfunction‐associated steatotic liver disease; MetALD, metabolic and alcohol‐related liver disease; MWA, microwave ablation; N, Node; R, Rest; RFA, Radiofrequency ablation; T, tumour; UICC, Union internationale contre le cancer.

^a^ Both active and former alcohol consumption are included in the LR group (data was self‐reported), 6 months of biochemically controlled sobriety were mandated for patients with ALD before wait‐listing for OLT.

^b^ Patients wait‐listed for OLT partly received surgical or interventional treatment prior to transplantation, resulting in a pathological T0 stage in the explanted liver specimen.

**FIGURE 2:**
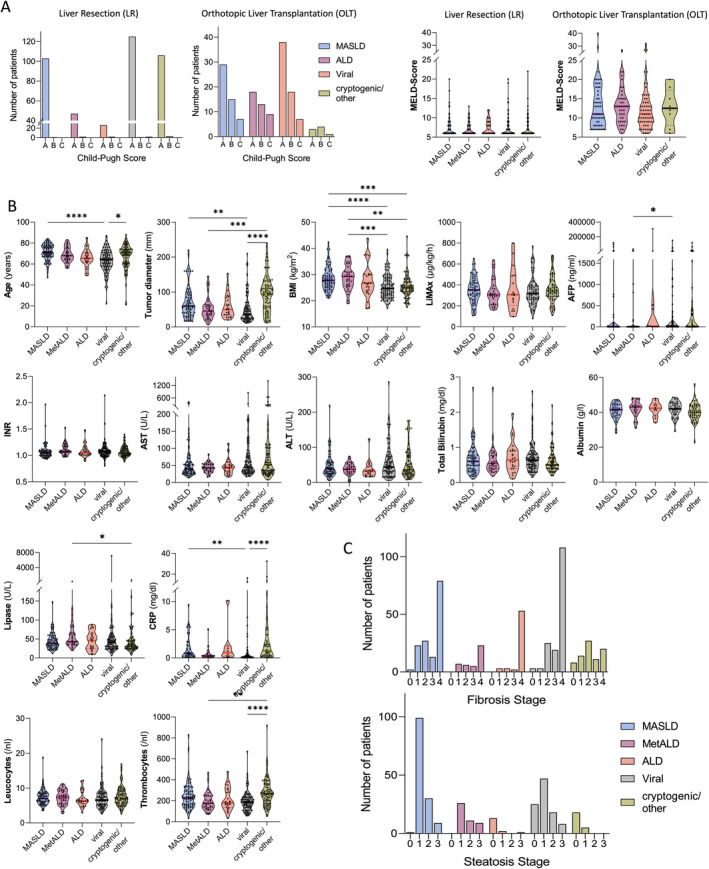
Predominant HCC aetiology and patient characteristics. (A) Liver disease severity, assessed by Child‐Pugh Score and MELD in the LR and OLT groups, divided by tumour aetiology. MELD was compared between aetiologies with Kruskal‐Wallis/Deniz Uluknn‘s multiple comparisons test. (B) Patient and laboratory parameters. Kruskal–Wallis test with significances given for Deniz Uluknn's multiple comparisons test of the LR/body composition cohort (*n* = 309). (C) Fibrosis and steatosis stage by HCC aetiology in the overall cohort. A total of *n* = 484 patients had a documented fibrosis stage and *n* = 322 had a documented steatosis stage of the non‐tumorous liver tissue. Friedman test with post hoc correction. Only significant comparisons are indicated with *p ≤ 0.05; **p ≤ 0.01; ***p ≤ 0.001; ****p ≤ 0.0001. AFP, α‐fetoprotein; ALD, alcohol‐related liver disease; ALT, alanine transaminase; AST, aspartate aminotransferase; BMI, body mass index; CRP, C‐reactive protein; LiMAx, liver maximum capacity test; LR, liver resection; MASLD, metabolic dysfunction‐associated steatotic liver disease; MetALD, metabolic and alcohol‐related liver disease; OLT, orthotopic liver transplantation; INR, international normalised ratio.

**FIGURE 3:**
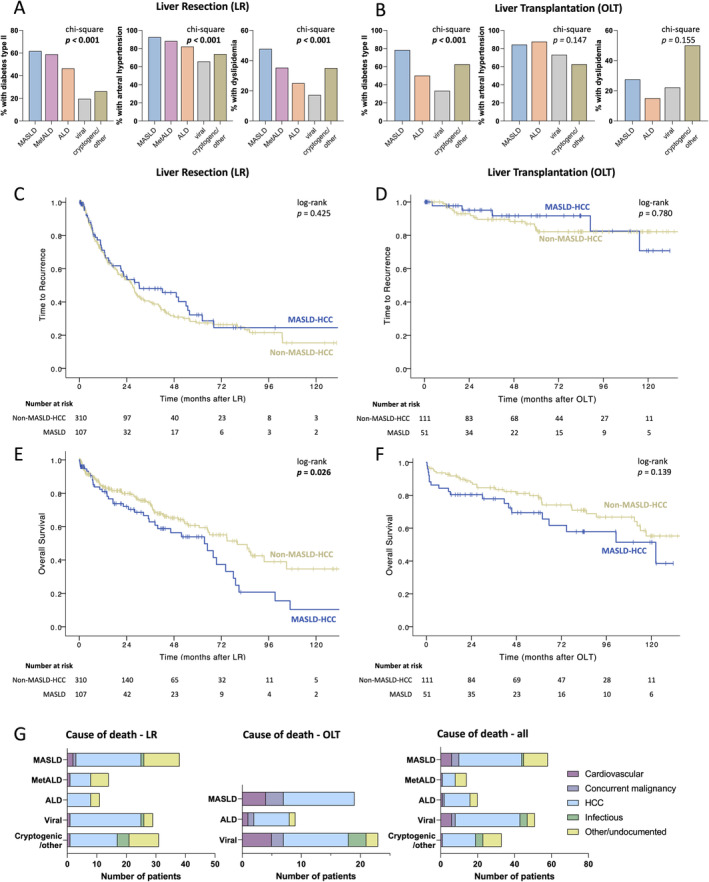
Distribution of cardiovascular risk factors and long‐term outcomes in patients with and without MASLD aetiology. Frequency (%) of cardiovascular risk factors among patients undergoing liver resection (MASLD *n* = 107, MetALD *n* = 51, ALD *n* = 28, viral *n* = 128, other/cryptogenic *n* = 103) (A) and transplantation (MASLD *n* = 51 ALD *n* = 40, viral *n* = 63, other/cryptogenic *n* = 8) (B). Pearson's Chi‐square *p* < 0.05 was considered significant. Time to recurrence (C, D) and overall survival (E, F) of patients with HCC, undergoing liver resection (C, E) or orthotopic liver transplantation (D, F). Log‐rank *p* < 0.05 was considered significant. (G) Causes of death, stratified by type of operation, with a total of *n* = 177 deaths and *n* = 52 deaths recorded in the LR and OLT cohorts, respectively. ALD, alcohol‐related liver disease; HCC, hepatocellular carcinoma; MASLD, metabolic dysfunction‐associated steatotic liver disease; MetALD, metabolic and alcohol‐related liver disease; LR, liver resection; OLT, orthotopic liver transplantation.

Patients undergoing OLT had higher Child–Pugh and MELD scores than patients undergoing liver resection, without significant differences between the underlying liver disease within groups (Figure 2A). No significant differences between the five HCC aetiologies were noted regarding the parameters of liver function and injury, such as the assessment of metabolic liver function by the liver maximum capacity test (LiMAx, Humedics GmbH Berlin, Germany) [30], transaminases, international normalised ratio (INR) and total bilirubin. Patients with MASLD and cryptogenic/other aetiology were significantly older and had significantly higher CRP values than patients with viral aetiology, while patients with viral aetiology had significantly higher AFP values than those with MetALD (Figure 2B). No significant association between HCC aetiology and the fibrosis or steatosis stage of the non‐tumorous resected tissue was noted when comparing all five aetiology groups (Figure 2C). The frequency of cardiovascular risk factors was significantly different between tumour aetiologies: Patients with MASLD, MetALD and ALD had the highest proportion of diabetes type II, dyslipidemia and arterial hypertension, particularly in the LR group (Chi‐square *p* < 0.001, Figure 3A). No significant differences in the frequency of cardiovascular risk factors were noted in the OLT population (Figure 3B).

Patients with MASLD were significantly older than non‐MASLD patients and had a higher BMI. Mean AFP was higher in MASLD patients undergoing LR but lower in MASLD patients undergoing OLT. Baseline tumour characteristics between MASLD patients and non‐MASLD patients were similar, except for a higher incidence of lymph node positivity in patients without MASLD undergoing liver resection (Table S1).

### 3.1 Oncologic Outcomes

Median follow‐up was 27 months (range: 0–140 months), with 194 (47%) cases of recurrence in the LT and 20 (12%) cases of recurrence recorded in the OLT group. Median TTR for patients after LR was 27 months (CI: 22.8—estimate not reached), and median TTR was not reached in patients undergoing OLT.

In the LR group, there were no significant differences between aetiologies regarding TTR (Figure S2A). Clinical factors associated with shorter TTR after LR were α‐fetoprotein (AFP) > 20 ng/mL, tumour diameter > 3 cm, intraoperative blood or plasma transfusions, portal vein thrombosis, R1 resection status, presence of lymphovascular or vascular invasion, tumour grading G3‐4, UICC stage 3–4 and pT stage T3‐4 (Table S2). Multivariable analysis identified R status (*p* = 0.002) and tumour grading G3‐4 (*p* = 0.006) as independently prognostic of TTR in LR, with both factors remaining significant after excluding in‐hospital mortality (Table 2).

**TABLE 2:** Multivariable analysis of time to recurrence and overall survival.

	Time to recurrence (TTR)		Overall survival (OS)	
	Relative risk (95% CI)	*p*	Relative risk (95% CI)	*p*
Patients after liver resection (*n* = 417)				
R status				
R0	1	0.002		
R1	2.396			
Grading				
G1‐2	1	0.006		
G3‐4	1.774			
Aetiology				
Non‐MASLD			1	0.019
MASLD			1.613 (1.083–2.404)	
Portal vein thrombosis				
No			1	< 0.001
Yes			2.996 (1.693–5.299)	
MELD score				
< 10			1	0.003
≥ 10			2.093 (1.285–3.411)	
Patients after liver resection, with patients with in‐hospital mortality excluded (*n* = 400)				
R status				
R0	1	0.002		
R1	2.389			
Grading				
G1‐2	1	0.006		
G3‐4	1.772			
Aetiology				
Non‐MASLD			1	0.016
MASLD			1.695 (1.104–2.602)	
Portal vein thrombosis				
No			1	0.002
Yes			2.785 (1.440–5.386)	
MELD score				
< 10			1	0.002
≥ 10			2.271 (1.337–3.858)	
All patients after liver transplantation (*n* = 162)				
UICC stage				
I–II	1	< 0.001	1	< 0.001
III–IV	16.162 (5.482–47.651)		6.105 (2.688–13.866)	
Tumour grading				
G1/G2	1	0.007		
G3/G4	4.175 (1.485–11.741)			
INR				
≤ 1.15			1	0.025
> 1.15			2.039 (1.092–3.808)	
Patients after liver transplantation, censored for in‐hospital mortality (*n* = 151)				
UICC stage				
I‐II	1	< 0.001	1	< 0.001
III‐IV	16.162 (5.482–47.651)		10.297 (4.295–24.688)	
Tumour grading				
G1/G2	1	0.007	1	0.025
G3/G4	4.175 (1.485–11.741)		2.449 (1.122–5.344)	
INR				
≤ 1.15			1	0.030
> 1.15			2.185 (1.081–4.419)	

*Note:* Significant variables from the Cox proportional hazards regression model are presented as hazard ratios (HR) and 95% confidence intervals (CI). Significant parameters in univariable analysis were included in the respective multivariable logistic regression model of TTR and OS. For TTR in the resection group, AFP (≤ 20 ng/mL/> 20 ng/mL), intraoperative blood transfusions, intraoperative FFP transfusions, R status, presence of lymphovascular invasion, tumour grading (G1‐2/G3‐4), presence of portal vein thrombosis, and UICC stage (1–2/3–4) were included. For TTR in transplanted patients, INR (≤ 1.15/> 1.15), tumour grading (G1‐2/ G3‐4) and UICC stage (1–2/ 3–4) were included. For OS in the resection group, MASLD criteria, haemoglobin (≤ 12/> 12 g/dL), intraoperative blood transfusions, R status (R0/R2), presence of lymphovascular invasion, tumour grading (G1‐2/G3‐4), presence of portal vein thrombosis, MELD Score (< 10/≥ 10), and UICC stage (1–2/3–4) were included. For OS in transplanted patients, Age (≤ 65/> 65), INR (≤ 1.15/> 1.15), tumour grading (G1‐2/G3‐4) and UICC stage (1–2/ 3–4) were included. To avoid multicollinearity with UICC stage, Tumour size, T stage and vascular invasion were omitted from the models.

Abbreviations: AFP, α‐fetoprotein; CI, confidence interval; G, grading; FFP, fresh frozen plasma; HR, hazard ratio; INR, international normalised ratio; MASLD, metabolic dysfunction‐associated steatotic liver disease; MELD, model for end‐stage liver disease; OS, overall survival; R, rest; TTR, time to recurrence; UICC, Union internationale contre le cancer.

In the OLT group, there was no significant difference in TTR between HCC aetiologies (*p* = 0.964) or in cases with vs. without histological steatosis (Figure S2B). Factors associated with shorter TTR were international normalised ratio (INR) > 1.15, vascular invasion, tumour grading G3‐4, UICC stage 3–4 and pT stage T3‐4 (Table S2). Of these, only UICC stage 3–4 (*p* < 0.001) and tumour grading G3‐4 (*p* = 0.007) were identified as independently prognostic for TTR and remained significant after censoring for in‐hospital mortality.

### 3.2 Overall Outcomes

A total of 125 (30%) deaths was recorded in the LR group and 52 (32%) deaths in the OLT cohort. Median OS after LR was 74.2 months (CI: 64.5–83.9), and median OS was 122 months (CI: 113–132) after OLT.

Significant differences in OS were noted, depending on the disease aetiology: Mean survival after LR (medians not reached for all groups) was the shortest in patients with MASLD (mean 57.8 months, CI: 46–69, median 63 months, CI: 43–84), followed by ALD (mean 58.8 months, CI: 40–68, median 37 months without CI reached) and MetALD (mean 64.4 months, CI: 53–75, median 66 months, CI: 26–123), while patients with cryptogenic/other (mean 70.0 months, CI: 55–85, median 74.7 months, CI: 26–123) and viral (mean 95.5 months, CI: 82–109, median survival not reached) aetiologies survived the longest (overall log‐rank *p* = 0.028, Figure S2C). While separating the cohort by MASLD and non‐MASLD aetiology was not significant for TTR (Figure 3C,D), OS after LR was significantly reduced in MASLD (median OS: 63 months, CI: 42–84; HR: 1.53, CI: 1.050–2.229) compared to non‐MASLD HCC aetiology (median OS 80 months, CI: 60–100, *p* = 0.026) (Figure 3E).

Clinical predictors of shorter OS after LR were tumour diameter > 3 cm, haemoglobin ≤ 12 g/dL, intraoperative blood transfusions, R1 status, lymphovascular and vascular invasion, portal vein thrombosis, MELD score, tumour grading G3‐4, UICC stage III–IV and pT stage T3–4 (Table S3). In multivariable analysis, MASLD aetiology (*p* = 0.019), portal vein thrombosis (*p* < 0.001) and MELD score ≥ 10 (*p* = 0.003) were identified as independent prognostic variables for OS after LR (Table 2), which remained significant even after excluding patients with in‐hospital mortality (MASLD *p* = 0.016, portal vein thrombosis and MELD score ≥ 10 both *p* = 0.002).

In patients with OLT, median OS of patients with MASLD was 102 months (CI: 58–187), with viral aetiology, 136 months (CI: 109–164), and 120 months (CI: estimates not reached) in patients with ALD and > 6 months sobriety (log rank *p* = 0.313, Figure S2D). There was a non‐significant trend towards inferior OS after OLT in patients with MASLD‐HCC (median not reached, *p* = 0.139, Figure 3F). Patients aged > 65 years at the time of OLT with INR > 1.15, tumour grading G3–4, UICC stage III–IV and pT stage T3–4 had a significantly shorter OS (Table S3). Multivariable analysis identified UICC stage III–IV (*p* < 0.001) and INR > 1.15 (*p* = 0.025) as independent factors for OS, and after excluding in‐hospital mortality, UICC stage III–IV (*p* < 0.001), tumour grading G3–4 (*p* = 0.025) and INR > 1.15 (*p* = 0.030) remained independently prognostic for OS in OLT (Table 2).

We next investigated the causes of death in the present cohort. Causes of death were categorised into cardiovascular, death from a concurrent malignancy (non‐HCC), death from HCC and infectious or other/undocumented (Figure 3G). The most prevalent cause of death in the cohort was from HCC, across all tumour aetiologies (108/177, 61%). Patients in the OLT group had a higher number of deaths from concurrent malignancy (6/52, 11.%), compared to that in the LR cohort (1/125, 0.8%). The frequency of the different causes of death did not differ significantly between patients with or without MASLD (Table S4).

### 3.3 Body Composition

A total of 309/417 (74%) LR patients had CT scans within 100 days prior to surgery available that were included in the body composition analysis. Pathological body composition (sarcopenia, sarcopenic obesity, myosteatosis and visceral obesity) was highly prevalent in this cohort but did not correlate with TTR or OS (Table S5).

**FIGURE 4:**
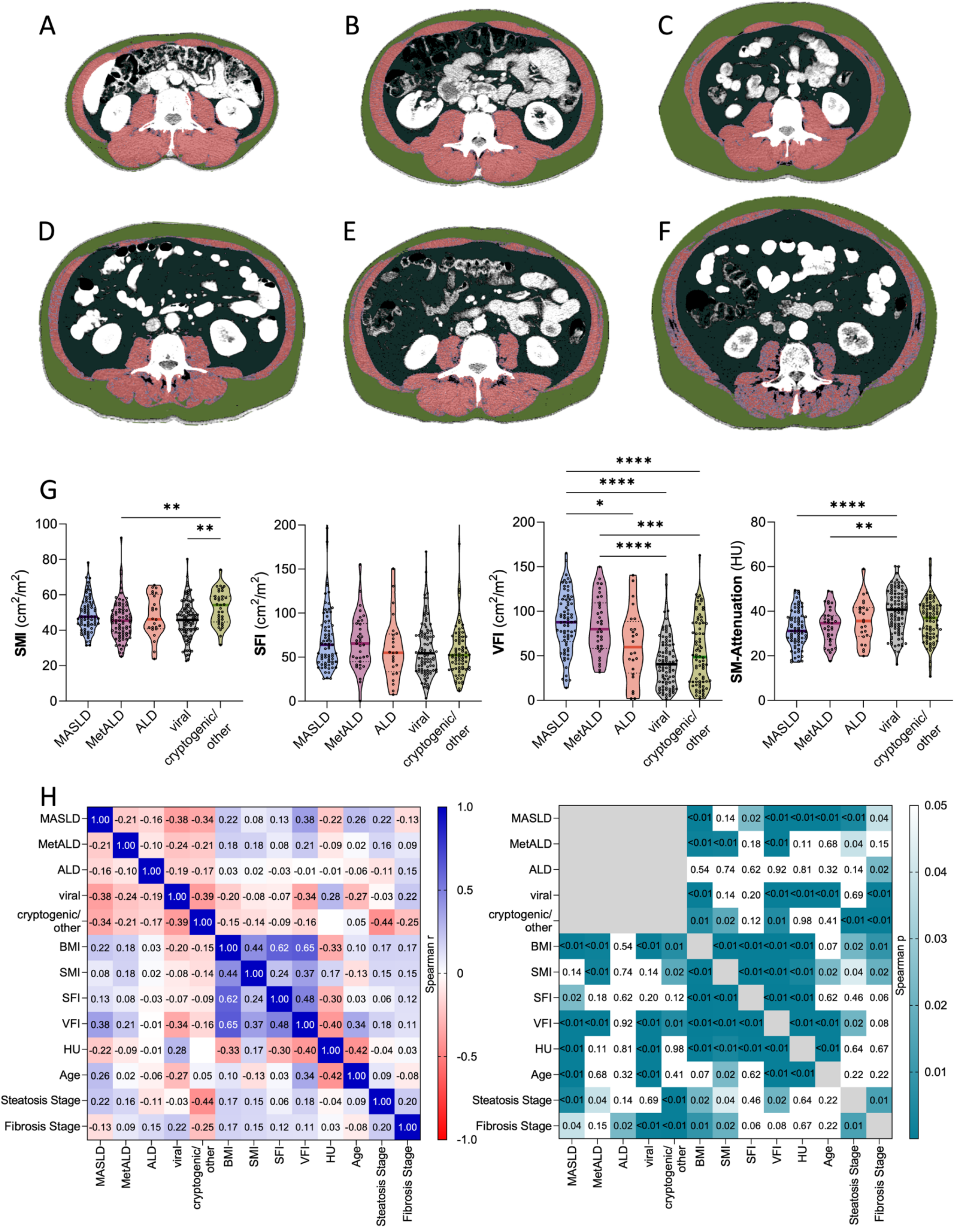
Association of HCC aetiology with body composition pathologies. CT‐based body composition assessment in patients with HCC, with lean muscle mass in red, fatty‐infiltrated muscle in violet, visceral fat in dark green, subcutaneous fat in light green. Representative images of typical body composition found in patients with viral aetiology: (A) Physiological/normal body composition (BMI: 21 kg/m^2^), (B) Physiological body composition (BMI: 26 kg/m^2^), (C) Visceral and subcutaneous obesity (increased light and dark green areas) with preserved muscle mass (BMI: 24 kg/m^2^). Representative images of patients with MetALD and MASLD: (D) Sarcopenia, sarcopenic obesity and visceral obesity with preserved muscle quality in a patient with MASLD (BMI: 25 kg/m^2^), (E) Isolated visceral obesity, muscle mass and quality preserved in a patient with MetALD (BMI: 26 kg/m^2^), (F) Myosteatosis (increased violet muscle area) and visceral obesity, with preserved overall muscle quantity (BMI: 28 kg/m^2^). (G) Skeletal muscle index (SMI), subcutaneous fat index (SFI), visceral fat index (VFI) and skeletal muscle (SM)‐attenuation in Hounsfield units (HU), with lower values representing a higher amount of intramuscular fat. Kruskall–Wallis test with significances given for Deniz Uluknn's multiple comparisons test of the LR/body composition cohort (*n* = 309). Only significant *p*‐values are indicated, with *p ≤ 0.05; **p ≤ 0.01; ***p ≤ 0.001; ****p ≤ 0.0001. (H) Correlation of HCC aetiology/underlying liver disease and body composition. Spearman's r (left) and p‐values (right) are given (*n* = 309). ALD, alcohol‐related liver disease; BMI, body mass index; CT, computed tomography; HCC, hepatocellular carcinoma; HU, Hounsfield units; MASLD, metabolic dysfunction‐associated steatotic liver disease; MetALD, metabolic and alcohol‐related liver disease; SFI, subcutaneous fat index; SMI, skeletal muscle index; VFI, visceral fat index.

When correlating body composition and disease aetiology, patients with MASLD and MetALD had distinct body composition hallmarks compared to patients with viral, cryptogenic and alcohol‐associated HCC aetiology (Figure 4A–F). Compared to patients without metabolic liver disease, patients with MASLD had a significantly higher incidence of myosteatosis, reflected in elevated skeletal muscle attenuation. The incidence of visceral and sarcopenic obesity was significantly higher in the MASLD group, with the VFI significantly higher than that in patients with ALD, viral and cryptogenic/other HCC aetiologies. The incidences of sarcopenia and subcutaneous obesity were comparable to non‐metabolic HCC. Patients with MetALD had a significantly higher incidence of visceral obesity than patients without metabolic liver disease and a trend towards a higher incidence of sarcopenia (95/201, 48% vs. 23/35, 66%, *p* = 0.05), with a significantly reduced SMI compared to the group with cryptogenic/other aetiologies. The rates of subcutaneous obesity and myosteatosis were similar to the group of non‐metabolic HCC aetiology (Table 3, Figure 4G). Patients with MASLD had the most significant changes of body composition, while viral aetiology correlated with the fibrosis grade (Figure 4H).

**TABLE 3:** Body composition and metabolic dysfunction‐associated HCC aetiology.

	Non‐metabolic aetiology (*n* = 201)	MetALD (*n* = 35)	*p* ^a^	MASLD (*n* = 74)	*p* ^a^
BMI (kg/m^2^)	25.0 (17.0–44.6)	29.3 (18.9–38.1)	**< 0.001**	27.9 (21.5–42.4)	**< 0.001**.
SMI (cm^2^/m^2^)	45.8 (22.7–92.2)	54.2 (31.7–74.2)	**0.001**	47.5 (31.5–78.2)	0.083
Sarcopenia, *n* (%)	104 (52.3)	12 (34.3)	0.050^b^	38 (51.4)	0.984^b^
Sarcopenic Obesity, *n* (%)	41 (20.4)	8 (22.9)	0.741^b^	25 (33.8)	**0.021** ^b^
VFI (cm^2^/m^2^)	44.6 (0.54–162.8)	79.9 (31.6–150.0)	**< 0.001**	87.7 (14.2–165.3)	**< 0.001**
Visceral obesity, *n* (%)	129 (60.0)	33 (94.3)	**< 0.001** ^b^	69 (93.2)	**< 0.001** ^b^
SFI (cm^2^/m^2^)	52.4 (2.7–178.6)	65.5 (21.7–155.3)	0.072	63.4 (25.5–240.3)	**0.017**
Subcutaneous obesity, *n* (%)	60 (30.2)	14 (40.0)	0.248^b^	29 (39.2)	0.157^b^
SM‐RA (HU), *n* (%)	38.8 (10.8–63.5)	34.6 (17.6–49.0)	**0.015**	31.2 (17.2–49.5)	**< 0.001**
Myosteatosis, *n* (%)	95 (47.5)	18 (51.4)	0.668^b^	46 (62.2)	**0.031** ^b^

*Note:* Body composition of 323 patients was analysed. Data presented as median and range, unless indicated otherwise. Bold values indicate a level of significance *p* < 0.05.

Abbreviations: BMI, body mass index; CA, carbohydrate antigen; CEA, carcinoembryonic antigen; CRP, C‐reactive protein; GGT, gamma glutamyl transferase; HU, Hounsfield Units; LiMAx, liver function maximum capacity test; MAFLD, metabolic dysfunction‐associated steatotic liver disease; MetALD, Metabolic and alcohol‐related liver disease; SFI, subcutaneous fat index; SMI, skeletal muscle index; SM‐RA, skeletal muscle radiation attenuation; VFA, Visceral fat area; VFI, Visceral fat index.

^a^ Based on Mann–Whitney *U* test compared to the “non‐metabolic aetiology” column, unless indicated otherwise.

^b^ Based on Pearson's chi‐square test between MASLD and non‐MASLD columns.

## 4 Discussion

While chronic hepatitis B virus infection still accounts for approximately 50% of all HCC cases worldwide, MASLD is rapidly emerging as the fastest growing aetiology of HCC in the Western world [31, 32]. While immunological exhaustion and auto‐reactivity constitute the major impediment to successful systemic checkpoint inhibitor therapy for advanced MASLD‐HCC [33], the survival of patients with early disease stages may be also impacted by accompanying non‐oncological factors, such as cardiovascular events and metabolic comorbidities [34]. As such, risk stratification for patients with MASLD‐HCC remains inadequate [35]. Only very few studies—both in the curative and palliative setting—analyse HCC aetiology beyond the traditional viral/non‐viral dichotomy that adequately described cohorts prior to the worldwide obesity epidemic. Here, we undertook a granular aetiological analysis of almost 600 patients over a decade of surgical HCC treatment, accounting for cardiovascular risk factors, body composition, clinico‐pathological data and prognosis. We focused on surgically treated patients in curative‐intent, without including patients undergoing ablation or non‐curative procedures.

Patients with MetALD‐ and MASLD‐HCC had a significantly higher incidence of fat‐associated body composition pathologies, while general characteristics were mostly similar between aetiology groups. Most prominently, patients with MetALD and MASLD had a significantly higher BMI and higher visceral fat content than patients with non‐metabolic aetiologies. Furthermore, patients with MASLD had a significantly higher amount of intramuscular fat, defined as myosteatosis. A higher proportion of MASLD patients had sarcopenic obesity. While in this and in other HCC cohorts [36], body composition pathologies did not have an isolated prognostic value, their detrimental role is well‐documented in other cohorts with end‐stage liver disease [25].

The finding that visceral, but not subcutaneous, fat was significantly increased in MetALD and MASLD is an important finding in the context of the newly proposed 2023 nomenclature [11] that merges the diagnosis of steatotic liver disease with cardiovascular risk factors. Increased visceral fat tissue represents a well‐known risk factor for type II diabetes, atherosclerosis and other cardiovascular morbidity, more so than BMI or subcutaneous obesity [37]. Storage and distribution of visceral fat are highly variable, even among individuals with similar BMI and subcutaneous fat deposits. In this context, exceeding the individual triglyceride‐storing capacity of the subcutaneous tissue in susceptible individuals is a major driver of visceral fat deposition [38]. Important effects of visceral obesity include hyperlipolytic metabolic and inflammatory activities, to which particularly the liver is exposed to through the drainage of blood from visceral adipose tissue into the portal vein, resulting in disrupted gluconeogenesis, inflammation and hepatocyte steatosis [37, 39]. The clinical relevance of hepatic steatosis has been recently demonstrated outside of the oncological context; in a group of adults with self‐reported pathological alcohol intake, individuals with steatosis (categorised as MetALD) had a higher risk of hepatic decompensation and overall mortality, compared to individuals without steatosis [40]. In HCC, mortality trends across curative and palliative HCC stages in the United States showed a shorter survival of patients with NAFLD‐HCC compared to viral hepatitis [41].

This study reinforces the new MASLD consensus definition from 2023 in the oncological context, evidencing an independent association of MASLD with OS in patients undergoing surgical treatment for HCC. This is underlined by our observation that hepatocyte steatosis alone had no prognostic value in the present resection and transplantation cohorts. An important argument in favour of the new MASLD definition was the heightened cardiovascular risk of patients with MASLD and thus the identification of a vulnerable group requiring interdisciplinary management. When analysing the causes of death across aetiologies, we did not observe a significant difference in cardiovascular deaths, with most patients dying from HCC. Patients with MASLD succumbed to similar causes of death as patients with non‐MASLD‐HCC, but these events occurred earlier than in the other HCC aetiologies.

Patients in the ALD group of this study had survival outcomes after LR that were comparably dismal to those from the MASLD group, despite not having a higher rate of recurrence. Variables that limit the interpretation of these data are the unknown status of postoperative sobriety in this cohort.

Further limitations of this study are linked to its retrospective nature: Alcohol consumption in the LR group was recorded on a self‐reported basis and was at a high risk for under‐reporting bias, typical for diseases with a high degree of associated stigma [42]. A challenge in applying the novel MetALD category to retrospective datasets lies in quantifying alcohol consumption beyond an ‘at‐risk’ amount. When MetALD was proposed, patients exceeding 50 g/d for women and 60 g/d for men were attributed to the ALD category, irrespective of steatosis and cardiometabolic risk factors. While the phenotypes of the ALD patients reported here and the one determined by quantifying daily alcohol consumption are most likely similar, our study underlines the need for categories that can be applied to retrospective datasets, which could be aided by future biomarkers or prospective phenotype characterisation. Similarly, an intriguing finding of the present aetiological categorisation is the large fraction of patients categorised as ‘cryptogenic’ in the LR but not in the OLT group. All patients diagnosed with HCC underwent aetiological workup to exclude treatable causes, such as viral hepatitis. However, unlike for liver transplantation listing, it was not mandatory to disclose alcohol consumption, and there was no obligation to undergo ALD‐specific laboratory testing. This limitation can be addressed in future prospective trials by embedding laboratory biomarkers for MetALD/ ALD as well as HCC into study protocols.

Patients wait‐listed for OLT had a mandated period of at least 6 months of absolute sobriety prior to transplantation—a variable that is not accounted for in the MetALD classification, and that prompted us to categorise these cases as (former) ALD. In this regard, the definition of MetALD in the OLT group was omitted because of patients' sobriety for over 6 months at the time of OLT and no recommendation for sober patients with MetALD in the consensus classification, so far. In the future, a better understanding of hepatic and systemic processes during periods of sobriety in end‐stage liver disease may aid the understanding on how these patients still share the metabolic risk factors that constitute the unique characteristics of the novel MetALD group. Similarly, our study was not able to evaluate steatotic liver disease longitudinally and to assess the level of steatosis before the tumour diagnosis. In this regard, longitudinal studies integrating body composition and biomarkers [43] are a valuable resource for a deeper understanding of the natural course of MASLD.

In conclusion, the present study explores a large collective of patients with HCC undergoing curative‐intend LR or OLT for HCC and provides evidence for detrimental non‐oncological outcomes in patients with MASLD. At the same time, the different categories of metabolic and alcohol‐associated liver disease did not confer differences in the recurrence of HCC. We demonstrate the independent prognostic value of the novel MASLD/MetALD consensus classification in a real‐life European cohort with HCC over a decade of HCC surgery.

## Author Contributions

**Deniz Uluk:** conceptualization, data curation, funding acquisition, investigation, methodology, project administration, software, supervision, writing – original draft, writing – review and editing. **Justus Pein:** data curation, formal analysis, investigation, visualization, writing – review and editing. **Sophia Herda:** data curation. **Frederik Schliephacke:** data curation, writing – review and editing. **Carolin V. Schneider:** formal analysis, writing – review and editing. **Jude Bitar:** data curation, software. **Katharina Dreher:** data curation. **Dennis Eurich:** resources. **Ingrid W. Zhang:** investigation, writing – review and editing. **Lukas Schaffrath:** data curation, investigation. **Timo A. Auer:** resources. **Federico Collettini:** resources. **Cornelius Engelmann:** resources. **Frank Tacke:** investigation, writing – review and editing. **Johann Pratschke:** resources. **Isabella Lurje:** conceptualization, formal analysis, investigation, methodology, supervision, visualization, writing – original draft, validation. **Georg Lurje:** conceptualization, funding acquisition, investigation, methodology, project administration, resources, supervision, writing – review and editing.

## Ethics Statement

This study was conducted in accordance with the Declaration of Helsinki, the good clinical practice (ICH‐GCP) guidelines and was approved by the institutional review board of the Charité—Universitätsmedizin Berlin, Berlin, Germany in 2021 (EA1/105/21).

## Consent

Informed consent was waived by the committee at study approval, based on the retrospective, pseudonymised analysis of available clinical patient data.

## Conflicts of Interest

G.L. reports receiving research funding and speakers' fees from Astellas Pharma, XVIVO, Bridge to Life, Organ recovery systems, Wyss Liver4Life, Orphalan and Aferetica S.R.L., and is on the advisory board of OrganOx, outside the submitted work. C.E. reports receiving an Else Kröner Fresenius Excellence Scholarship (German Research Foundation, DFG) and an EU‐Horizon grant, has shares with UCL Spin‐off company Hepyx Ltd., has received consulting fees from and is on the advisory board of Albireo/Ipsen and Boehringer Ingelheim, lecture honoraria/ travel support from Gilead and Albireo/Ipsen. F.T. reports research funding to his institution from AstraZeneca, MSD, Gilead, Agomab, consulting fees from AstraZeneca, Gilead, GSK, Abbvie, Alnylam, BMS, Intercept, Inventiva, Pfizer, Novartis, Novo Nordisk, MSD, Sanofi, lecture honoraria/travel support from Gilead, AbbVie, Falk, Merz, Intercept, Sanofi, Astra Zeneca, Orphalan, and is on the advisory board of Sanofi and Pfizer. The remaining authors have no conflicts of interest to declare.

## Supporting information


Data S1.

## Data Availability

Due to European and federal law for data protection and due to the sensitive nature of the results, patient's data were not shared in a public repository. Data and methodology will be made available upon reasonable request to the corresponding author.
