# Supplementary material for: Metabolic Dysfunction‐Associated Steatotic Liver Disease (MASLD) Impacts Long‐Term Outcomes After Curative‐Intent Surgery for Hepatocellular Carcinoma
Source: Aliment Pharmacol Ther. 2025 Feb 18;61(8):1318–32. doi: 10.1111/apt.70002 (PMC11950813; doi:10.1111/apt.70002)
Supplement: Supplementary file 1 — Data S1. [file APT-61-1318-s001.docx]

**Supplementary Material**

**Metabolic dysfunction-associated steatotic liver disease (MASLD) impacts long-term outcomes after curative-intent surgery for hepatocellular carcinoma.**

**Supplementary Table 1**: Baseline characteristics between MASLD and non-MASLD patients

|  | **Liver resection** (n=417) | | | **Orthotopic liver transplantation** (n=162) | | |
| --- | --- | --- | --- | --- | --- | --- |
|  | **MASLD** (n=107) | **Non-MASLD** (n=310) | ***p*=^†^** | **MASLD** (n=51) | **Non-MASLD** (n=111) | ***p*=^†^** |
| Age (years) | 70.1±10.9 | 64.6±13.9 | **<.001** | 62.2±6.0 | 60.2±6.4 | **.043** |
| BMI (kg/m^2^) | 30.2±21.7 | 26.4±5.1 | **<.001** | 30.9±5.9 | 27.2±5.3 | **<.001** |
| LiMAx (μg/kg/h) | 351.9±118.1 | 338.2±137.6 | .129 | 173.2±96.6 | 188.2±121.3 | .819 |
| Tumor diameter (mm) | 64.7±41.6 | 62.6±44.5 | .270 | 25.1±12.3 | 27.0±19.1 | .693 |
| AFP (ng/ml) | 4319.8± 18787.0 | 4220.5±  24919.2 | **.013** | 113.9±707.1 | 181.7±727.9 | **.013** |
| Lymph node positivity  n, (%) | 0 (0.0) | 11 (3.5) | **.032** | 0 (0.0) | 0 (0.0) | n.a. |
| Lymphovascular invasion n, (%) | 7 (6.5) | 18 (5.8) | .800 | 1 (1.9) | 2 (1.8) | .931 |
| Vascular invasion,  n (%) | 22 (20.6) | 80 (25.8) | .289 | 4 (7.8) | 11 (9.9) | .700 |
| Perineural invasion,  n (%) | 0 (0.0) | 5 (1.6) | .248 | 0 (0.0) | 0 (0.0) | n.a. |

Data presented as mean ± Standard deviation if not noted otherwise.

**^†^** Mann-Whitney U Test was used for continuous variables, Chi Square Test or two-sided Fisher’s exact test for categorical variables.

Abbreviations: AFP, α-fetoprotein; BMI, body mass index; MASLD, metabolic dysfunction-associated steatotic liver disease.

**Supplementary Table 2**: Univariable analysis of time to recurrence (TTR) by clinico-pathological characteristics

|  | **Liver resection** (n=417) | | | | **Orthotopic liver transplantation** (n=162) | | | |
| --- | --- | --- | --- | --- | --- | --- | --- | --- |
|  | **n (%)** | **Median TTR (95%CI)** | **Hazard Ratio (95% CI)** | ***p*=**^†^ | **n (%)** | **Median TTR (95%CI)** | **Hazard Ratio (95% CI)** | ***p*=**^†^ |
| **Sex** |  |  |  |  |  |  |  |  |
| Male | 310 (74.3) | 27  (22.6-31.5) |  | .881 | 136 (84.0) | n.a. |  | .216 |
| Female | 107 (25.7) | 25  (8.7-41.5) |  |  | 26 (16.0) | n.a. |  |  |
| **Age** (years) |  |  |  |  |  |  |  |  |
| ≤ 65 | 164 (34.8) | 23  (15.2-30.1) |  | .241 | 115 (71.0) | n.a. |  | .870 |
| > 65 | 253 (53.7) | 28  (23.2-32.8) |  |  | 47 (29.0) | n.a. |  |  |
| **α-Fetoprotein** (AFP) | | | | | | | | |
| ≤ 20 ng/mL | 179 (42.9) | 29  (18.2-40.3) | 1 | **.041** | 119 (70.4) | n.a. |  | .262 |
| >20 ng/mL | 121 (29.0) | 13 (4.1-21.5) | 1.409 (1.013-1.961) |  | 41 (25.3) | n.a. |  |  |
| **Tumor diameter** (in cm, on preoperative imaging) | | | | | | | | |
| ≤ 3 | 104 (24.9) | 46  (26.4-65.8) | 1 | **.036** | 116 (71.6) | n.a. |  | .056 |
| > 3 | 312 (74.8) | 22  (16.8-28.1) | 1.429  (1.022-1.998) |  | 46 (28.4) | n.a. |  |  |
| **International normalized ratio** (INR) | | | | | | | | |
| ≤ 1.15 | 333 (79.9) | 28  (22.4-33.3) |  | .240 | 45 (27.8) | n.a. | 1 | **.036** |
| > 1.15 | 84 (20.1) | 27 (20.8-32.9) |  |  | 117 (72.2) | n.a. | 2.489 (1.031-6.013) |  |
| **Total Bilirubin,** mg/dl | |  |  |  |  |  |  |  |
| ≤ 1.2 | 374 (89.7) | 26 (21.7-31.2) |  | .539 | 74 (45.7) | n.a. |  | .083 |
| > 1.2 | 31 (7.4) | 28 (14.2-42.3) |  |  | 88 (54.3) | n.a. |  |  |
| **Albumin,** g/l |  |  |  |  |  |  |  |  |
| ≤ 42 | 125 (30.0) | 23 (11.1-34.2) |  | .190 | 126 (77.8) | n.a. |  | .779 |
| > 42 | 95 (22.8) | 28 (20.8-34.9) |  |  | 15 (9.3) | n.a. |  |  |
| **Hemoglobin,** g/dl | | |  |  |  |  |  |  |
| ≤ 12 | 88 (21.1) | 27 (21.1-33.0) |  | .063 | 69 (42.6) | n.a. |  | .938 |
| > 12 | 327 (78.4) | 27 (21.6-32.4) |  |  | 93 (57.4) | n.a. |  |  |
| **MELD score** |  |  |  |  |  |  |  |  |
| < 10 | 353 (84.7) | 26 (20.5-31.9) |  | .852 |  |  |  |  |
| ≥ 10 | 52 (12.5) | 28 (25.0-30.9) |  |  |  |  |  |  |
| **Intraoperative blood transfusions** | | |  |  |  |  |  |  |
| No | 337 (80.8) | 29 (23.1-35.5) | 1 | **.011** | 22 (13.6) | n.a. |  | .509 |
| Yes | 79 (18.9) | 14 (3.2-24.4) | 1.563 (1.106-2.208) |  | 140 (86.4) | n.a. |  |  |
| **Intraoperative FFP transfusions**^‡^ | | | |  |  |  |  |  |
| No | 223 (53.4) | 30 (20.6-40.2) | 1 | **.021** |  |  |  |  |
| Yes | 192 (46.0) | 22 (16.0-28.1) | 1.395 (1.051-1.851) |  |  |  |  |  |
| **R status**^‡^ |  |  |  |  |  |  |  |  |
| R0 | 363 (87.1) | 29 (21.9-35.3) | 1 | **<.001** |  |  |  |  |
| R1/Rx | 40 (9.6) | 13 (5.4-20.2) |  |  |  |  |  |  |
| **Lymphovascular Invasion** | | |  |  |  |  |  |  |
| No | 385 (92.3) | 28 (23.2-32.8) | 1 | **.006** | 158 (97.5) | n.a. |  | .161 |
| Yes | 25 (5.9) | 7 (3.6-10.4) | 2.174 (1.231-3.838) |  | 3 (1.8) | n.a. |  |  |
| **Microvascular invasion** | |  |  |  |  |  |  |  |
| No | 313 (75.1) | 31 (22.4-39.9) | 1 | **<.001** | 146 (90.1) | n.a. | 1 | **.006** |
| Yes | 102 (24.5) | 14 (9.6-18.0) | 2.069 (1.524-2.828) |  | 15 (9.3) | n.a. | 3.733 (1.351-10.312) |  |
| **Portal vein thrombosis** | |  |  |  |  |  |  |  |
| No | 377 (90.4) | 28 (23.0-33.0) | 1 | **<.001** | 160 (98.8) | n.a. |  | .631 |
| Yes | 34 (8.2) | 11 (3.2-19.7) | 2.069 |  | 2 (1.2) | n.a. |  |  |
| **Tumor grading** |  |  |  |  |  |  |  |  |
| G1 / G2 | 317 (76.0) | 29.3 (23.2-35.3) | 1 | .**003** | 118 (72.8) | n.a. | 1 | **.003** |
| G3 / G4 | 77 (18.5) | 12.6 (8.1-17.2) | 1.629 (1.185-2.323) |  | 26 (16.0) | n.a. | 3.787  (1.481-9.687) |  |
| **Tumor stage (UICC)** | | |  |  |  |  |  |  |
| I / II | 343 (82.3) | 23.3 (23.0-35.6) | 1 | **<.001** | 152 (93.8) | n.a. | 1 | **<.001** |
| III /IV | 72 (17.3) | 12.4 (10.2-14.7) | 1.958 (1.379-2.780) |  | 9 (5.6) | 26.3 (0-52.6) | 11.590 (4.306-31.192) |  |
| **pT stage** |  |  |  |  |  |  |  |  |
| pT1-2 | 345 (82.7) | 29.3 (23.0-35.6) | 1 | **<.001** | 151 (93.2) | n.a. | 1 | **<.001** |
| pT3-4 | 65 (15.6) | 11 (3.6-17.6) | 2.555 (1.779-3.668) |  | 9 (5.6) | 26 (0-66.2) | 9.544 (3.381-26.938) |  |
| **Histological Steatosis** (>5% of hepatocytes) | | | |  |  |  |  |  |
| no | 40 (9.6) | 20.8 (7.1-34.4) |  | .204 | 22 (13.5) | n.a. |  | .783 |
| yes | 222 (53.2) | 29.3 (18.1-40.5) |  |  | 81 (50.0) | n.a. |  |  |

^†^Based on log rank test.

^‡^Only 2 patients undergoing transplantation did not receive FFP transfusions, and no patient undergoing liver transplantation had R1 status, therefore survival outcomes were not programmed for this group.

n.a. – estimates not reached.

Abbreviations: CI, confidence interval; FFP, fresh frozen plasma; MELD, model for end-stage liver disease; OS, overall survival; pT, pathological Tumor stage; R, rest; TTR, time to recurrence; UICC, Union internationale contre le cancer.

**Supplementary Table 3**: Univariable analysis of overall survival (OS) by clinico-pathological characteristics

|  | **Liver resection** (n=417) | | | | **Orthotopic liver transplantation** (n=162) | | | |
| --- | --- | --- | --- | --- | --- | --- | --- | --- |
|  | **n (%)** | **Median OS (95%CI)** | **Hazard Ratio (95% CI)** | ***p=***^†^ | **n (%)** | **Median OS (95%CI)** | **Hazard Ratio (95% CI)** | ***p=***^†^ |
| **Sex** |  |  |  |  |  |  |  |  |
| Male | 310 (74.3) | 69  (60.8-78.3) |  | .139 | 136 (84.0) | 122  (116.2-128.5) |  | .158 |
| Female | 107 (25.7) | 87  (62.6-111.4) |  |  | 26 (16.0) | n.a. |  |  |
| **Age** (years) |  |  |  |  |  |  |  |  |
| ≤ 65 | 164 (34.8) | 66  (42.7-89.3) |  | .842 | 115 (71.0) | 136  (113.7-159.2) | 1 | **.024** |
| > 65 | 253 (53.7) | 74  (62.5-85.9) |  |  | 47 (29.0) | 101  (59.3-143.0) | 1.924  (1.077-3.434) |  |
| **α-Fetoprotein** (AFP) | | | | | | | | |
| ≤ 20 ng/mL | 179 (42.9) | 78  (61.6-94.6) |  | .337 | 119 (70.4) | 114 (97.6-130.7) |  | .143 |
| >20 ng/mL | 121 (29.0) | 75 (48.0-101.8) |  |  | 41 (25.3) | 136 (72.2-200.6) |  |  |
| **Tumor diameter** (in cm, on preoperative imaging) | | | | | | | | |
| ≤ 3 | 104 (24.9) | 93  (60.0-127.4) | 1 | **.013** | 116 (71.6) | n.a. |  | .144 |
| > 3 | 312 (74.8) | 65  (46.6-83.1) | 1.735 (1.117-2.694) |  | 46 (28.4) | 112 (64.7-160.2) |  |  |
| **International normalized ratio** (INR) | | | | | | | | |
| ≤ 1.15 | 333 (79.9) | 75  (63.1-86.8) |  | .093 | 45 (27.8) | 117  (33.0-201.4) | 1 | **.009** |
| > 1.15 | 84 (20.1) | 64.9 (35.1-94.6) |  |  | 117 (72.2) | 136  (105.1-167.8) | 2.073 (1.186-3.622) |  |
| **Total Bilirubin,** mg/dl | |  |  |  |  |  |  |  |
| ≤ 1.2 | 374 (89.7) | 69 (59.1-80.0) |  | .958 | 74 (45.7) | 117 (91.2-143.2) |  | .160 |
| > 1.2 | 31 (7.4) | 81 (0.0-177.8) |  |  | 88 (54.3) | 136 (105-167.8) |  |  |
| **Albumin,** g/l |  |  |  |  |  |  |  |  |
| ≤ 42 | 125 (30.0) | 66 (24.6-107.4) |  | .096 | 126 (77.8) | 136 (110.2-162.7) |  | .853 |
| > 42 | 95 (22.8) | 74 (61.6-86.8) |  |  | 15 (9.3) | n.a. |  |  |
| **Hemoglobin,** g/dl | | |  |  |  |  |  |  |
| ≤ 12 | 88 (21.1) | 43 (12.5-73.7) | 1 | **.017** | 69 (42.6) | 114 (92.5-135.7) |  | .742 |
| > 12 | 327 (78.4) | 78 (65.3-90.9) | 1.596 (1.082-2.354) |  | 93 (57.4) | 136 (n.a.) |  |  |
| **MELD** score |  |  |  |  |  |  |  |  |
| < 10 | 353 (84.7) | 78 (68-87.8) | 1 | .001 | 49 (30.2) | 122 (108.3-136.4) |  | .364 |
| ≥ 10 | 52 (12.5) | 37 (16.6-57.2) | 2.103 (1.333-3.317) |  | 111 (68.5) | 136 (105.1) |  |  |
| **Intraoperative blood transfusions** | | |  |  |  |  |  |  |
| No | 337 (80.8) | 75 (63-86.4) | 1 | **.002** | 22 (13.6) | 122 (n.a.) |  | .295 |
| Yes | 79 (18.9) | 75 (13.1-136.7) | 1.872 (1.239-2.829) |  | 140 (86.4) | 136 (108-164.9) |  |  |
| **Intraoperative FFP transfusions**^‡^ | | | |  |  |  |  |  |
| No | 223 (53.4) | 80 (72.8-87.4) |  | .092 |  |  |  |  |
| Yes | 192 (46.0) | 65 (53.0-76.7) |  |  |  |  |  |  |
| **R status**^‡^ |  |  |  |  |  |  |  |  |
| R0 | 363 (87.1) | 74 (63.0-86.8) | 1 | **.032** |  |  |  |  |
| R1 | 40 (9.6) | 39 (0.0-84.2) |  |  |  |  |  |  |
| **Lymphovascular Invasion** | | |  |  |  |  |  |  |
| No | 385 (92.3) | 74 (63.2-86.3) | 1 | **<.001** | 158 (97.5) | 122 (113-131.8) |  | .602 |
| Yes | 25 (5.9) | 14 (0-30.2) | 2.994 |  | 3 (1.8) | n.a. |  |  |
| **Microvascular invasion** | |  |  |  |  |  |  |  |
| No | 313 (75.1) | 79 (65.3-93.1) | 1 | **.002** | 146 (90.1) | n.a. | 1 | **.007** |
| Yes | 102 (24.5) | 53 (20.6-86.1) | 1.841 |  | 15 (9.3) | 55 (0-111.2) | 2.552 (1.258-5.177) |  |
| **Portal vein thrombosis** | |  |  |  |  |  |  |  |
| No | 377 (90.4) | 75 (63.4-86.4) | 1 | **<.001** | 160 (98.8) | 122 (112.9-131.9) |  | .675 |
| Yes | 34 (8.2) | 10 (4.3-15.5) | 3.957 |  | 2 (1.2) | 2 (n.a.) |  |  |
| **Tumor grading** |  |  |  |  |  |  |  |  |
| G1 / G2 | 317 (76.0) | 78 (67.9-88.4) | 1 | .**020** | 118 (72.8) | 136 (111.1-161.8) | 1 | **.021** |
| G3 / G4 | 77 (18.5) | 53 (34.5-72.2) | 1.621 (1.076-2.443) |  | 26 (16.0) | 65 (0-133.0) | 2.151  (1.107-4.181) |  |
| **Tumor stage (UICC)** | | |  |  |  |  |  |  |
| I / II | 343 (82.3) | 78.1 (66.9-89.4) | 1 | **<.001** | 152 (93.8) | 136 (113.5-159.4) | 1 | **<.001** |
| III /IV | 72 (17.3) | 67 (4.5-51.0) | 2.498 (1.677-3.720) |  | 9 (5.6) | 72.1 (23.7-30.1) | 4.458 (2.069-9.607) |  |
| **pT stage** |  |  |  |  |  |  |  |  |
| pT1-2 | 345 (82.7) | 79 (65.8-92.6) | 1 | **<.001** | 151 (93.2) | 136 (113.5-159.4) | 1 | **<.001** |
| pT3-4 | 65 (15.6) | 22 (0.0-45.3) | 3.044 (2.0-4.631) |  | 9 (5.6) | 26 (18.4-35.4) | 4.164 (1.853-9.359) |  |
| **Histological Steatosis** (>5% of hepatocytes) | | | | |  |  |  |  |
| no | 40 (9.6) | n.a. |  | .585 | 22 (13.5) | n.a. |  | .180 |
| yes | 222 (53.2) | 69.5 (58.0-81.1) |  |  | 81 (50.0) | 117 (102.3-132.1) |  |  |

^†^Based on log rank test.

^‡^Only 2 patients undergoing transplantation did not receive FFP transfusions, and no patient undergoing liver transplantation had R1 status, therefore survival outcomes were not programmed for this group.

n.a. – estimates not reached.

Abbreviations: CI, confidence interval; FFP, fresh frozen plasma; MELD, Model for end-stage liver disease, OS, overall survival; pT, pathological tumor stage; R, rest; TTR, time to recurrence; UICC, Union internationale contre le cancer.

**Supplementary Table 4**: Causes of death (COD) compared between MASLD and non-MASLD patients

|  | **Liver resection** (n=417) | | | **Orthotopic liver transplantation** (n=162) | | |
| --- | --- | --- | --- | --- | --- | --- |
|  | **MASLD** (n=107) | **Non-MASLD** (n=310) | ***p*=^†^** | **MASLD** (n=51) | **Non-MASLD** (n=111) | ***p*=^†^** |
| Cardiovascular COD  n, (%) | 2 (1.9) | 3 (1.0) | .606 | 4 (7.8) | 6 (5.4) | .726 |
| Concurrent malignancy n, (%) | 1 (0.9) | 0 (0.0) | .257 | 3 (5.8) | 3 (2.7) | .380 |
| HCC  n (%) | 22 (20.6) | 55 (17.7) | .517 | 12 (23.5) | 19 (17.1) | .335 |
| Infectious COD  n (%) | 1 (0.9) | 5 (1.6) | .611 | 0 (0.0) | 3 (2.7) | .552 |
| Other COD/ undocumented  n (%) | 14 (13.1) | 22 (7.1) | .057 | 0 (0.0) | 2 (1.8) | 1.000 |

**^†^** Chi Square Test or two-sided Fisher’s exact test.

Abbreviations: COD, cause of death; HCC, hepatocellular carcinoma; MASLD, metabolic dysfunction-associated steatotic liver disease.

**Supplementary Table 5:** Body composition and outcome in patients with HCC undergoing liver resection.

|  | **n=309 (%)** | **Median TTR (95%CI)** | ***p*=**^†^ | **Median OS (95%CI)** | ***p*=**^†^ |
| --- | --- | --- | --- | --- | --- |
| **Sarcopenia** | | | | | |
| No | 154 (49.8) | 27 (21.3-33.6) | .489 | 75 (61.5-88.0) | .887 |
| Yes | 154 (49.8) | 18 (13.1-23.8) |  | 86 (38.3-133.8) |  |
| **Sarcopenic obesity** | | | | | |
| No | 236 (76.4) | 24 (15.2-32.1) | .725 | 75 (62.0-87.5) | .198 |
| Yes | 74 (23.9) | 26 (16.2-36.2) |  | n.a. |  |
| **Visceral obesity** | | | | | |
| No | 87 (28.2) | 19.2 (3.3-35.1) | .710 | 105 (17.4-192.7) | .627 |
| Yes | 222 (71.8) | 24 (3.0-30.0) |  | 75 (62.2-87.2) |  |
| **Subcutaneous obesity** | |  |  |  |  |
| No | 205 (66.3) | 22 (13.7-30.4) | .445 | 80 (67.0-93.2) | .629 |
| Yes | 104 (33.7) | 26 (4.8-35.7) |  | 74 (n.a.) |  |
| **Myosteatosis** | | | | | |
| No | 150 (48.5) | 22 (10.6-33.5) | .988 | 81 (57.7-104.2) | .350 |
| Yes | 159 (51.5) | 24 (16.5-30.8) |  | 75 (45.3-104.1) |  |

Body composition of patients with available preoperative CT scans.
^†^based on log rank test

n.a. – estimates not reached

Abbreviations: CI, confidence interval; OS, Overall survival; TTR, Time to recurrence

**Supplementary Figures**

**
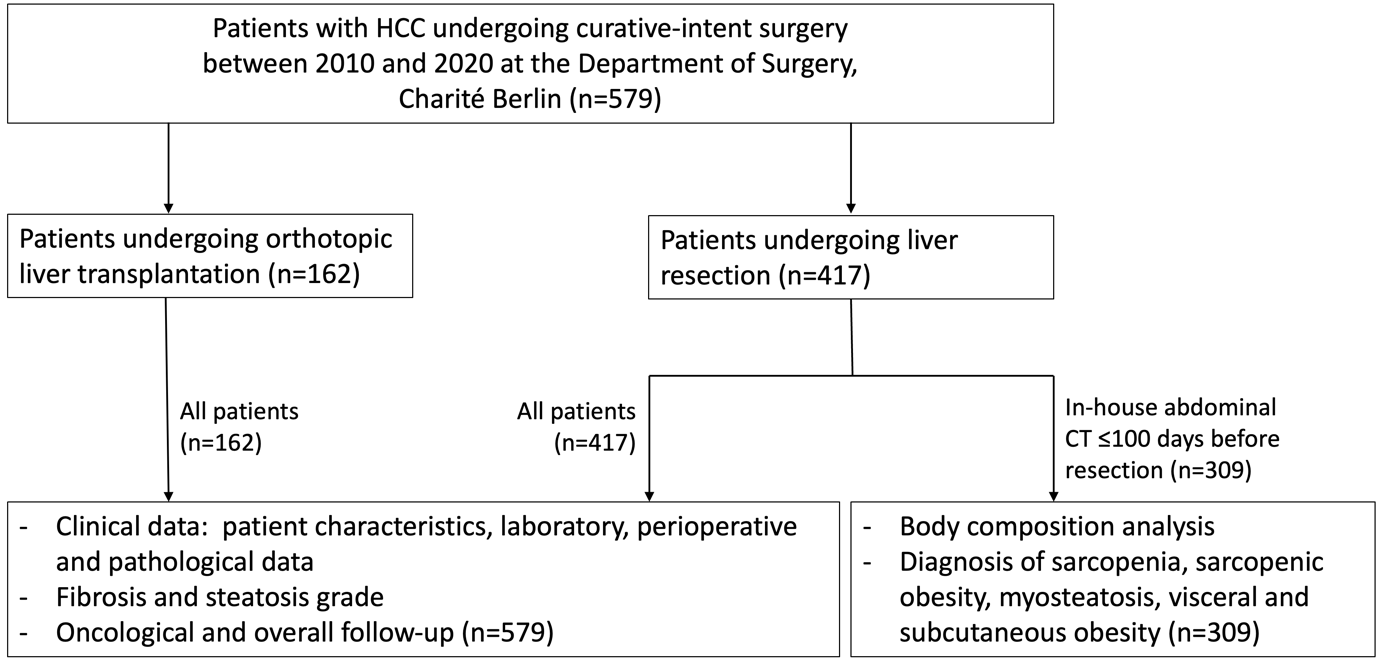
**

**Supplementary Figure 1**: Flow chart of in- and exclusion criteria.

Abbreviations: CT, computed tomography; HCC, hepatocellular carcinoma.

**
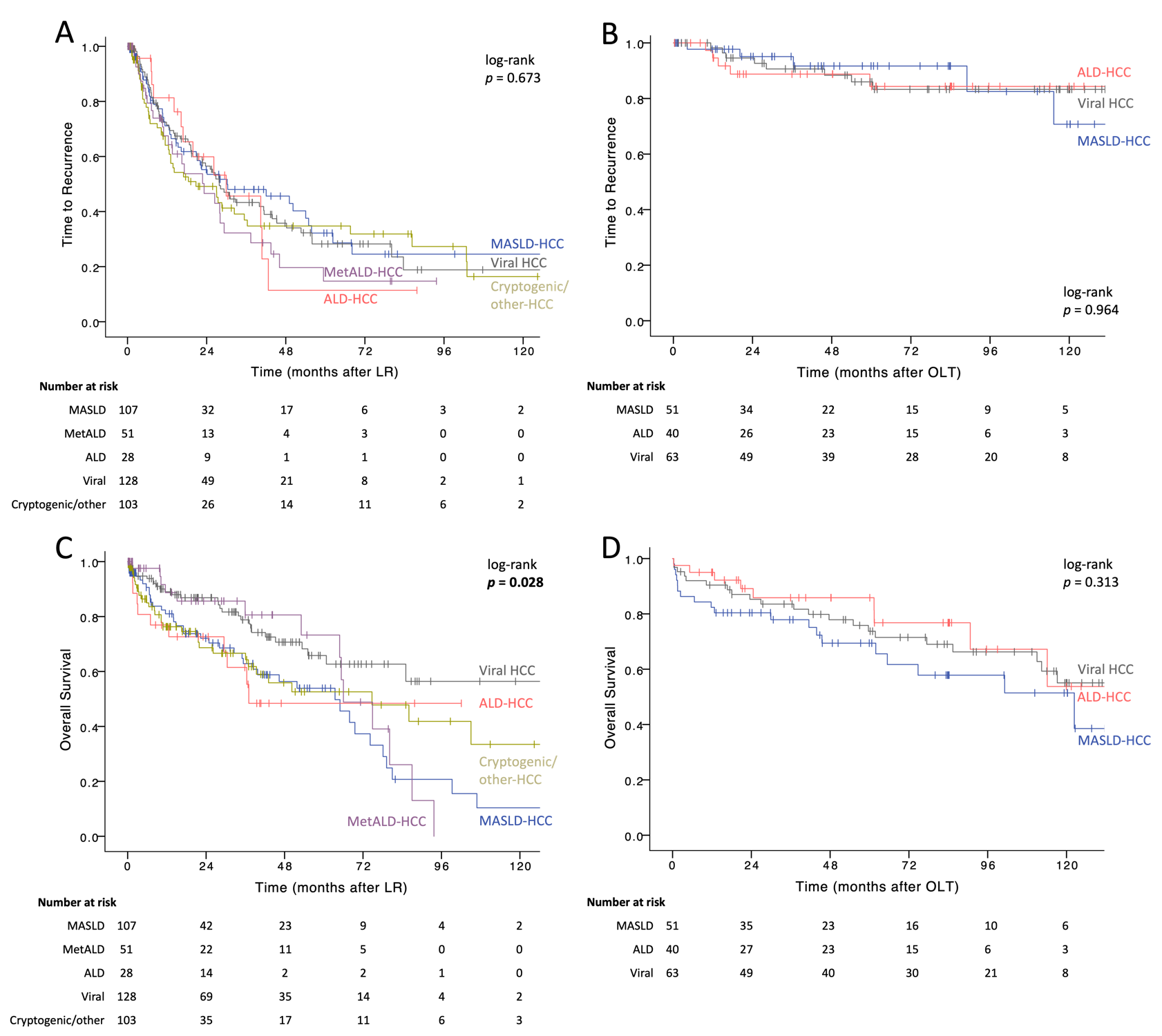
**

**Supplementary Figure 2**: Long-term oncological and overall outcomes, stratified by HCC etiology.

Due to the low number of patients (n=8) and events (n=2 for TTR and OS) in the cryptogenic/other etiology group in patients undergoing OLT, data was not plotted for this etiology in panels B and D. In the OLT group, patients with ALD-HCC were mandated 6 months of sobriety prior to wait-listing.

Abbreviations: ALD, alcohol-related liver disease; HCC, hepatocellular carcinoma; LR, liver resection; MASLD, metabolic dysfunction-associated steatotic liver disease; MetALD, metabolic and alcohol related liver disease; OLT, orthotopic liver transplantation
